# Supplementary material for: Particle Phagocytosis Amplifies Secretion of Small Extracellular Vesicles in Macrophages
Source: bioRxiv. 2026 Feb 2:2026.01.29.701815. Preprint. [Version 1] doi: 10.64898/2026.01.29.701815 (PMC12889446; doi:10.64898/2026.01.29.701815)
Supplement: Supplement 1 [file media-1.pdf]

Supplemental Information for:

Particle Phagocytosis Amplifies Secretion of Small Extracellular Vesicles in Macrophages

Michael Trautmann-Rodriguez,[1] Vilina Akala,[2] Nicole A. Gill,[1] Emma R. Sudduth,[1] Catherine A. Fromen[1,2*]

[1]Department of Chemical and Biomolecular Engineering, University of Delaware, Newark, DE 19716

[2]Department of Biomedical Engineering, University of Delaware, Newark, DE 19716

Author ORCIDs:

Trautmann-Rodriquez (0000-0002-2965-3537)

Sudduth (0000-0002-8141-6416)

Fromen (0000-0002-7528-0997)

*corresponding author

Catherine A. Fromen

cfromen@udel.edu

150 Academy St.

Newark, DE 19716

(302) 831-3649

Table of Contents:

	Page
**Supplemental Table S1:** Antibody table for flow cytometry experiments	S3
**Supplemental Figure S1:** Example flow cytometry gating for RAW264.7, BMMs, and THP-1	S4
**Supplemental Figure S2:** Example flow cytometry gating for BALF isolated alveolar macrophage from *in vivo* mice studies	S4
**Supplemental Figure S3:** Comparison between densities of PEGDA(-)50 and PEGDA(-)75 particle chemistries	S5
**Supplemental Figure S4:** Second SEM imaging panel of BMMs dosed with distinct particle chemistry	S5
**Supplemental Figure S5:** Second TEM imaging panel of BMMs dosed with distinct particle chemistry	S6
**Supplemental Figure S6:** Image analysis of vesicle per cell in TEM imaging of particle-dosed BMMs	S6
**Supplemental Figure S7:** Volcano plots for whole cell and secreted proteomic analysis	S7
**Supplemental Figure S8:** Hierarchical clustering heat map for significantly differentiated proteins in UT, PEGDA(-)50, and LPS-dosed BMMs whole cell proteomic analysis	S7
**Supplemental Figure S9:** Metabolic activity of PEGDA(-)50 and EV^{MO-NP} dosed BMMs using CellTiter Glo 2.0	S8
**Supplemental Figure S10:** Confirmation of pro-inflammatory BMM phenotype after 72 hrs incubation	S9
**Supplemental Figure S11:** Confirmation of anti-inflammatory BMM phenotype after 72 hrs incubation	S10
**Supplemental Figure S12:** Example flow gating of Cy5 expression in phagocytosis-driven BMM EVs after 72 hrs of incubation	S11
**Supplemental Figure S13:** Example flow cytometry gating for BMMs and CD11b marker	S11

Supplemental Table S1: Antibodies for flow cytometry analysis. Antibodies for staining and magnetic bead capture were purchased through Biolegend with corresponding fluorophore, clone number, and dilution factor listed.

Marker of Interest	Distributor	Fluorophore	Clone	Dilution
CD11b	Biolegend	Pacific Blue	M1/70	1:100
CD80	Biolegend	Pacific Blue	16-10A1	1:100
MHC II	Biolegend	BV 785	M5/114.15.2	1:100
CD163	Biolegend	BV 421	S15049I	1:100
Siglec-F	BD Pharmingen	APC-Cy7	E50-2440	1:100
CD63	Biolegend	NA	NVG-2	NA
CD9	Biolegend	NA	MZ3	NA
ICAM-1	Biolegend	NA	YN1/1.7.4	NA

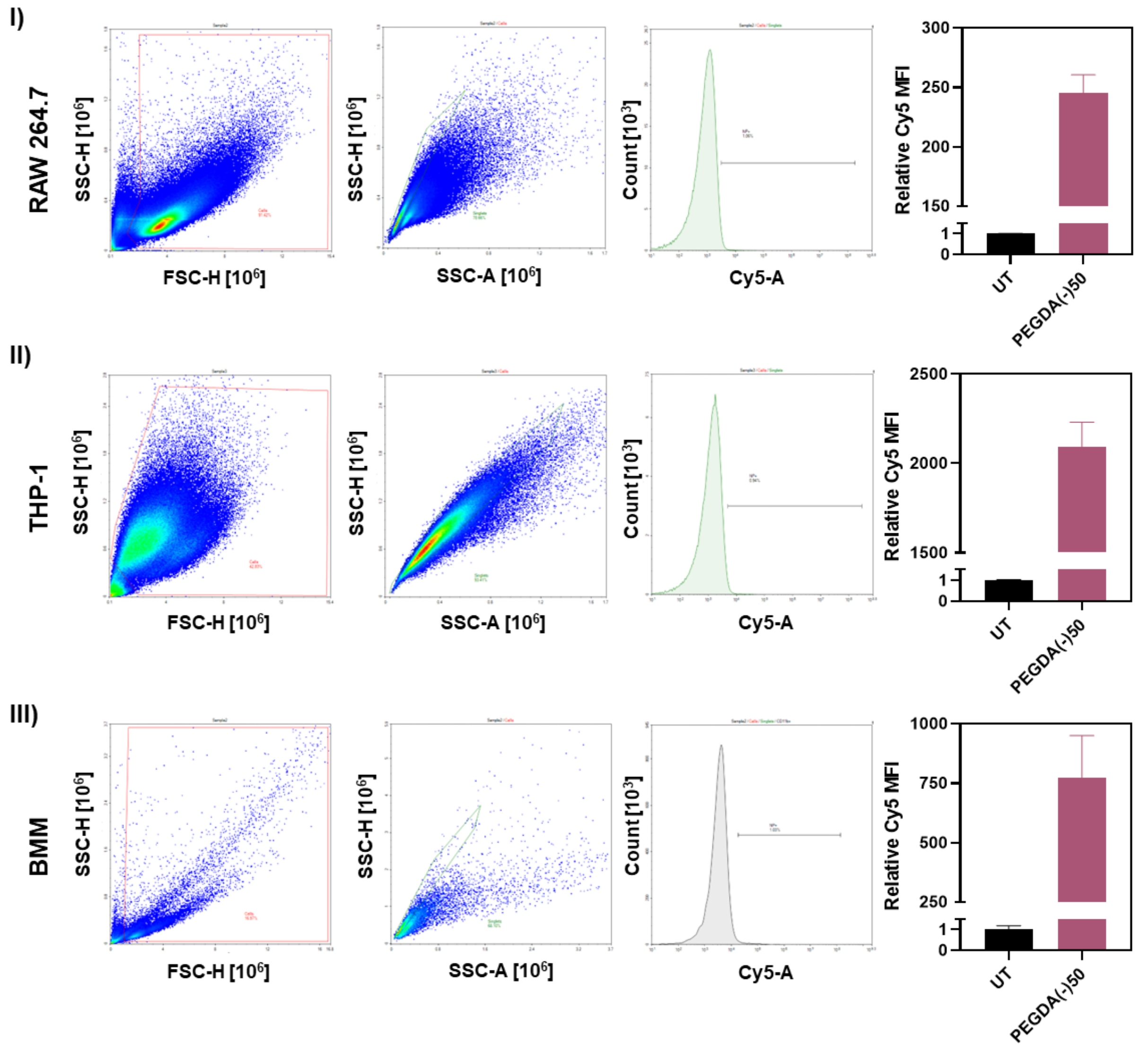

Supplemental Figure S1 Example flow gating (I) for RAW264.7, (II)THP-1, and (III) BMM cultures with Cy5 MFI for untreated and PEGDA(-)50 dosed with (100 µg/mL) cultures.

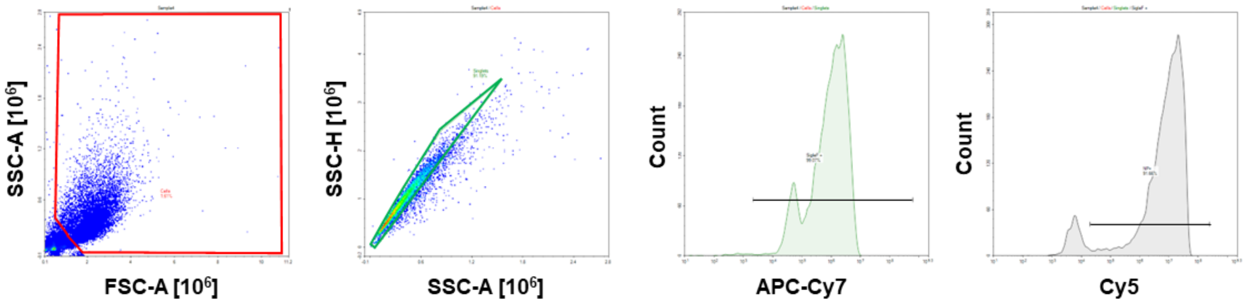

Supplemental Figure S2. Example flow gating for alveolar macrophages isolated from PEGDA(-)50-dosed mice. Density plots selecting for cells, singlets, and Siglec-F+, and showing PEGDA(-)50 internalization.

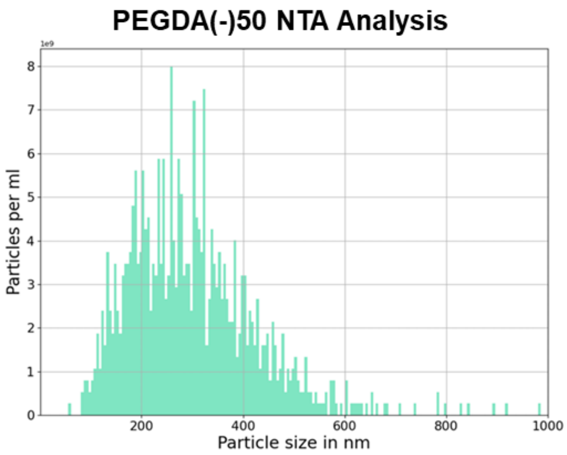

Particle Chemistry	Diameter (nm)	Particle count (particle/mL)	TGA Mass (µg)	Volume (nm^3)	PEGDA Mass /particle (mg/particle)	Particle PEGDA Density (kg/m^3)
PEGDA(-)50	283.3	2.59e11	52	9.52e7	4.02e-12	42.16
PEGDA(-)75	277.4	3.93e11	155	8.94e7	7.86e-12	87.88

Supplemental Figure S3. NTA and TGA of PEGDA(-)50 and PEGDA(-)75 chemistry, used to calculate particle density based on diameter, stock particle counts, and stock concentration. Volume assumed to be spherical.

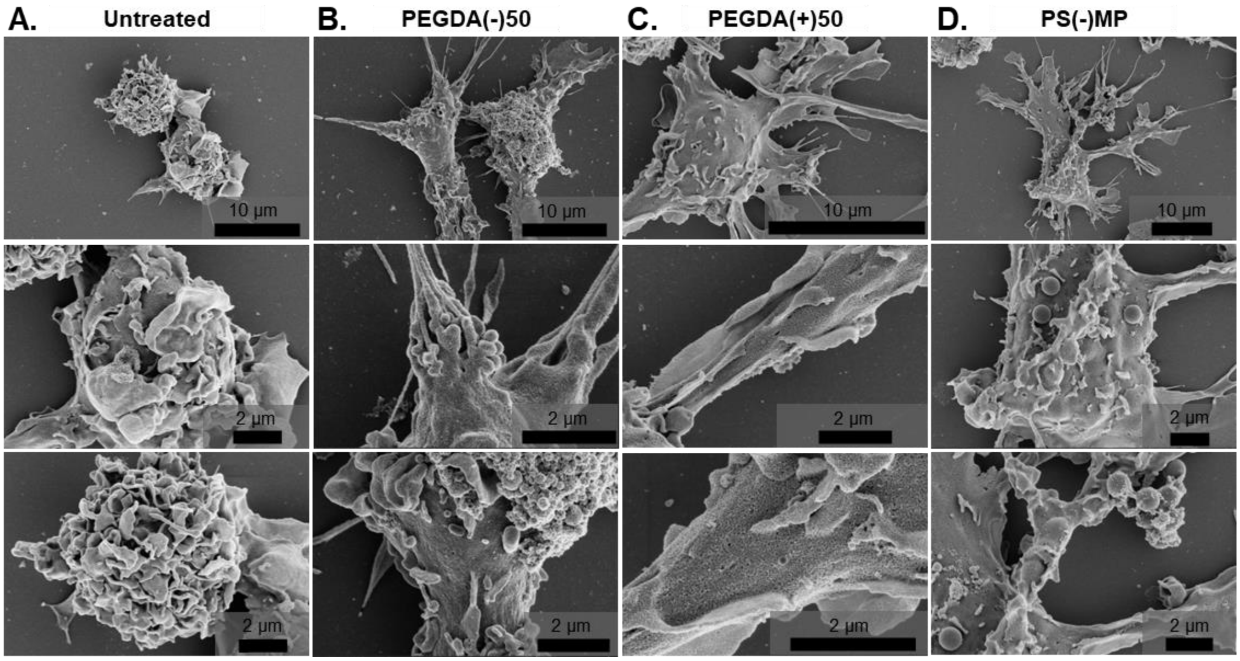

Supplemental Figure S4. Replicate set of SEM images for **A)** untreated, **B)** PEGDA(-)50, **C)** PEGDA(+)50, and **D)** PS(-)MP BMM cultures.

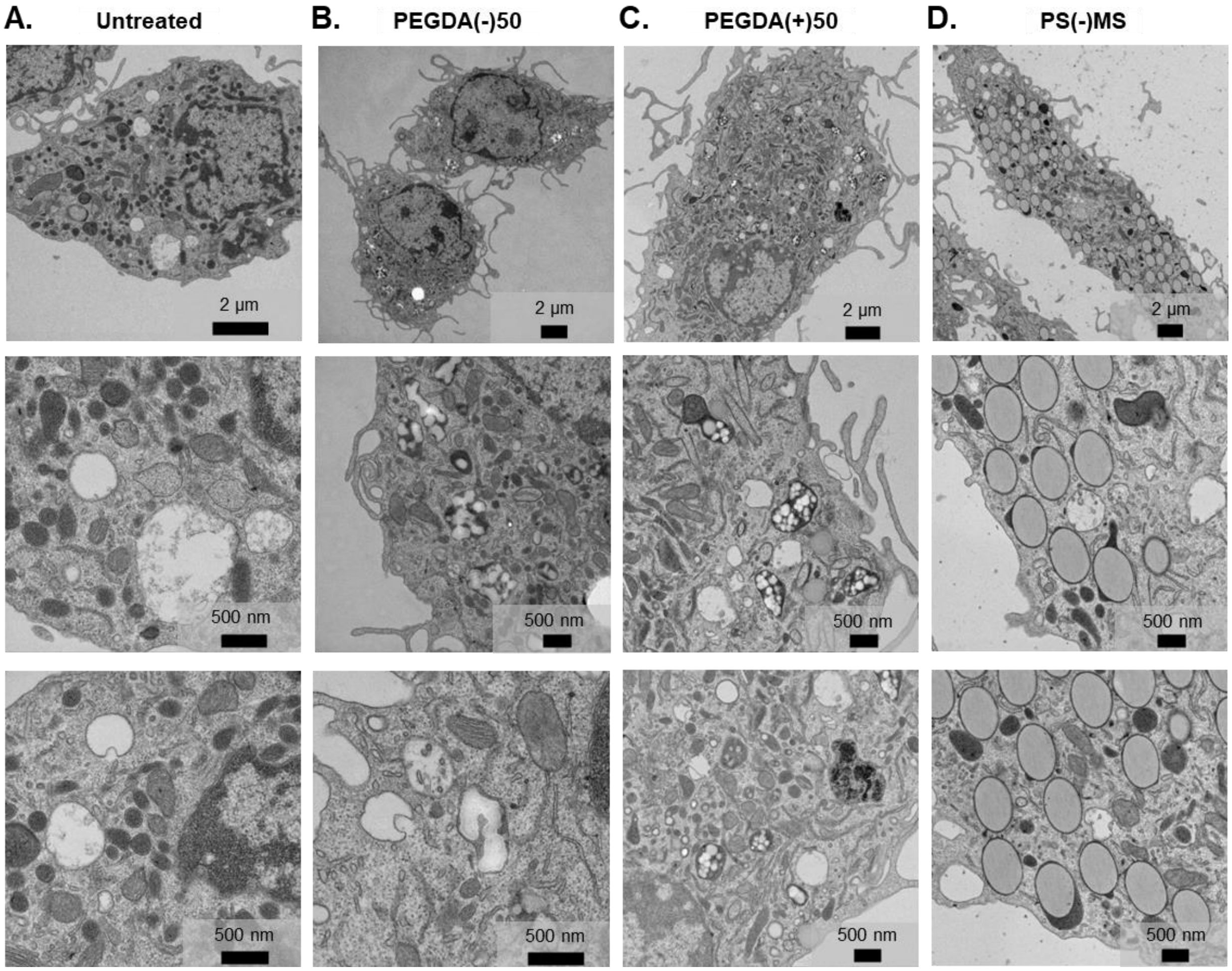

Supplemental Figure S5. Replicate set of TEM images for **A)** untreated, **B)** PEGDA(-)50, **C)** PEGDA(+)50, and **D)** PS(-)MP BMM cultures.

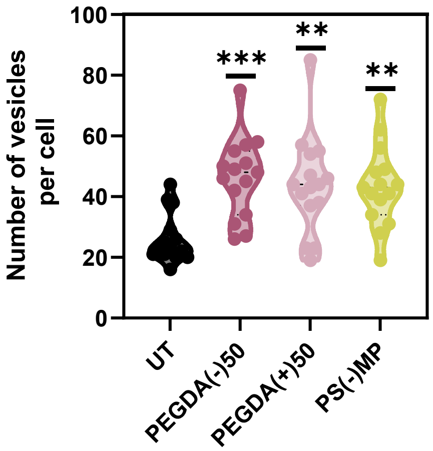

Supplemental Figure S6. Graph showing the number of vesicles in each cell, as calculated from the TEM images, PEGDA(-)50(p-value) = 0.0005, PEGDA(+)50(p-value) = 0.0025, and PS(-)MP(p-value) = 0.0031 (ordinary one-way ANOVA with a Turkey's multiple comparison test, N = 15).

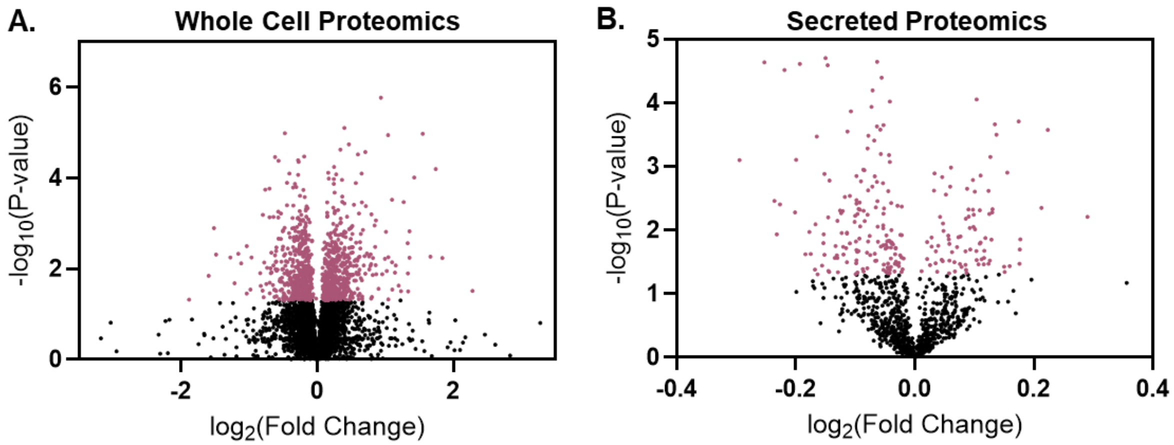

Supplemental Figure S7. Volcano plots for identified proteins in **A)** whole cell proteomic study and **B)** secreted proteomics study.

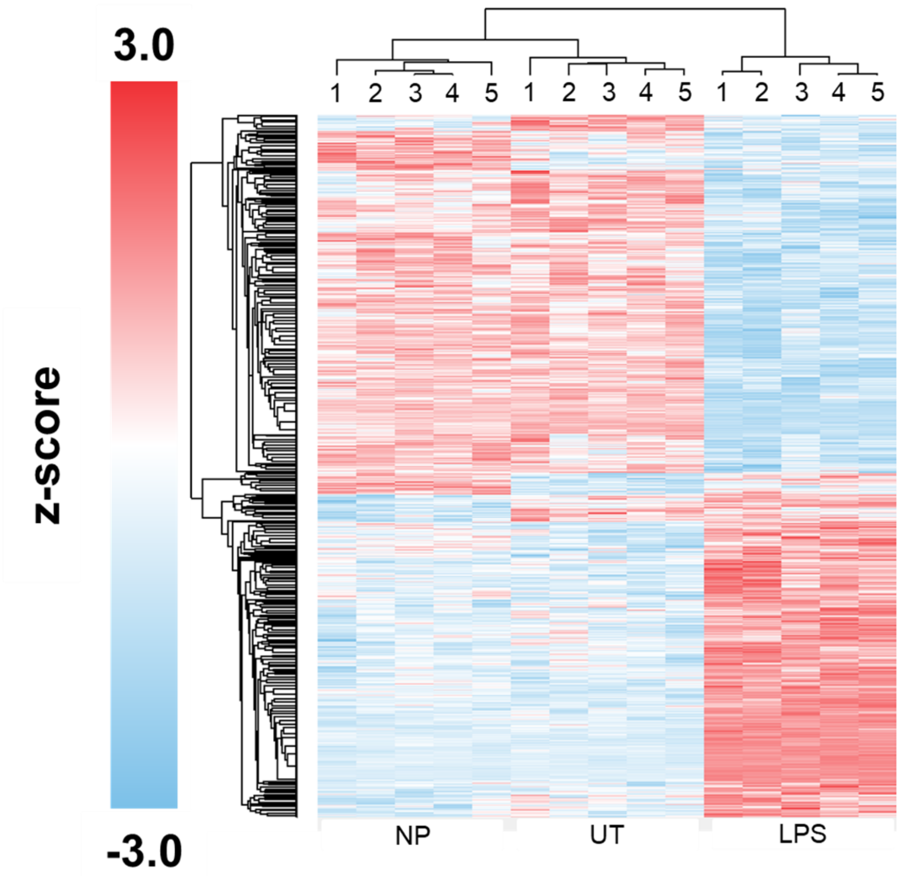

Supplemental Figure S8. Hierarchical clustering of proteomic datasets comparing protein expression in UT, PEGDA(-)50-dosed, and LPS-dosed BMMs. Clustering highlights similarity between UT and NP, when compared to inflammatory (LPS-dosed) BMMs.

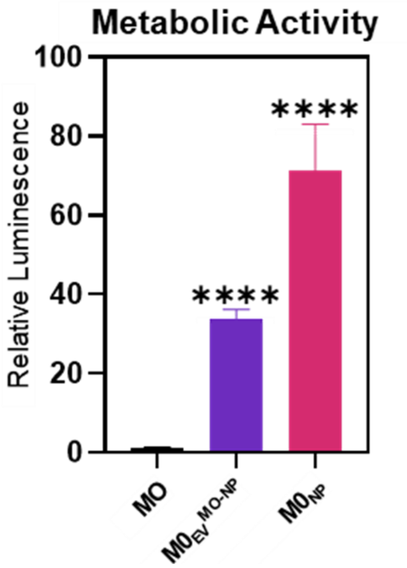

Supplemental Figure S9. Metabolic activity of BMM cultures after 4 days of dosing with PEGDA PEGDA(-)50 and NP derived EVs as measured through Cell Titer glo 2.0 (N = 4, SD error bars) ****$p<0.0001$, using Brown-Forsythe and Welch ANOVA tests.

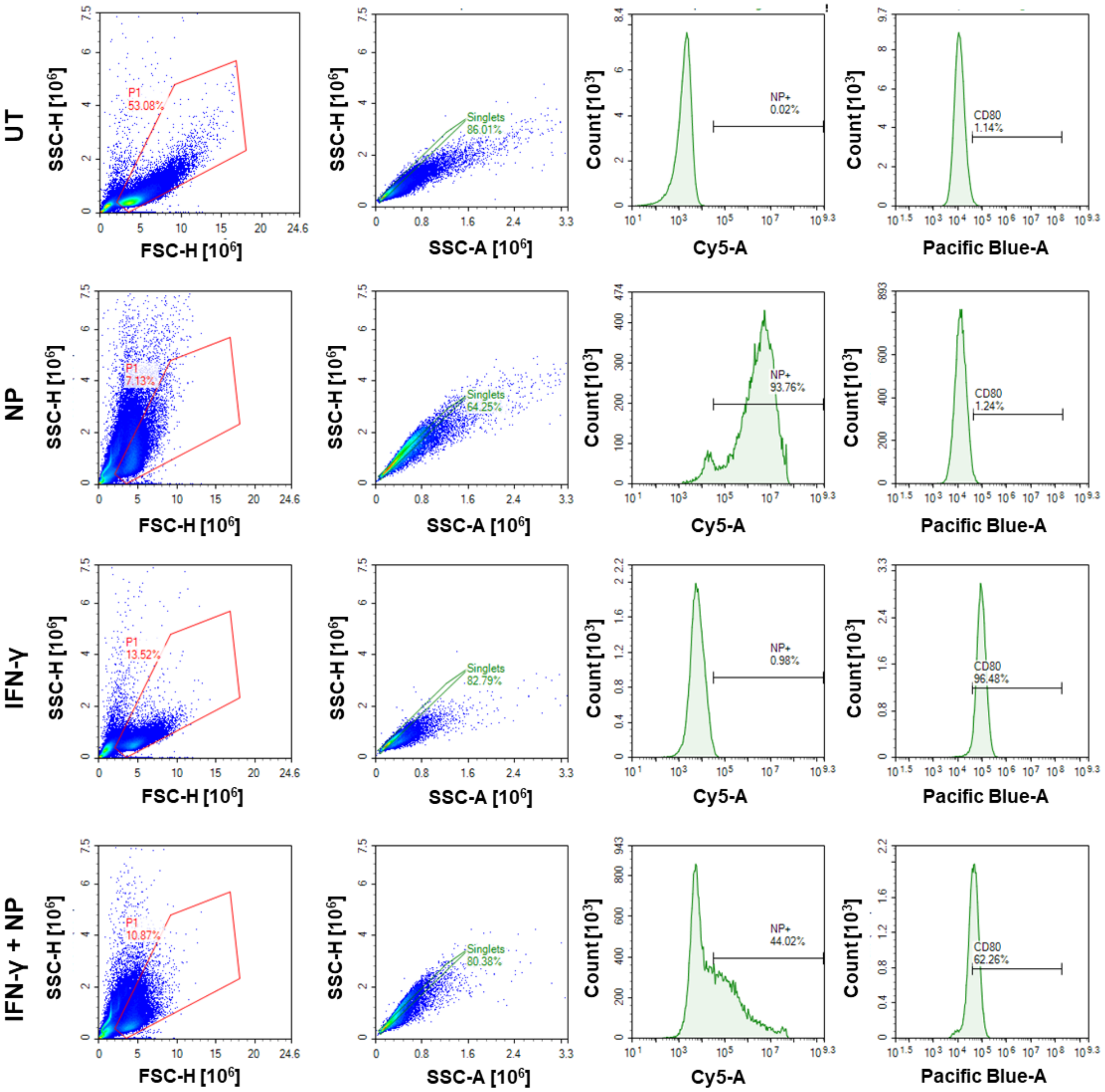

Supplemental Figure S10. Example flow cytometry plots of BMM samples following dosing and 72 hrs incubation with PEGDA(-)50, IFN-γ, or PEGDA(-)50 and IFN-γ. Pro-inflammatory marker CD80 highlighted here.

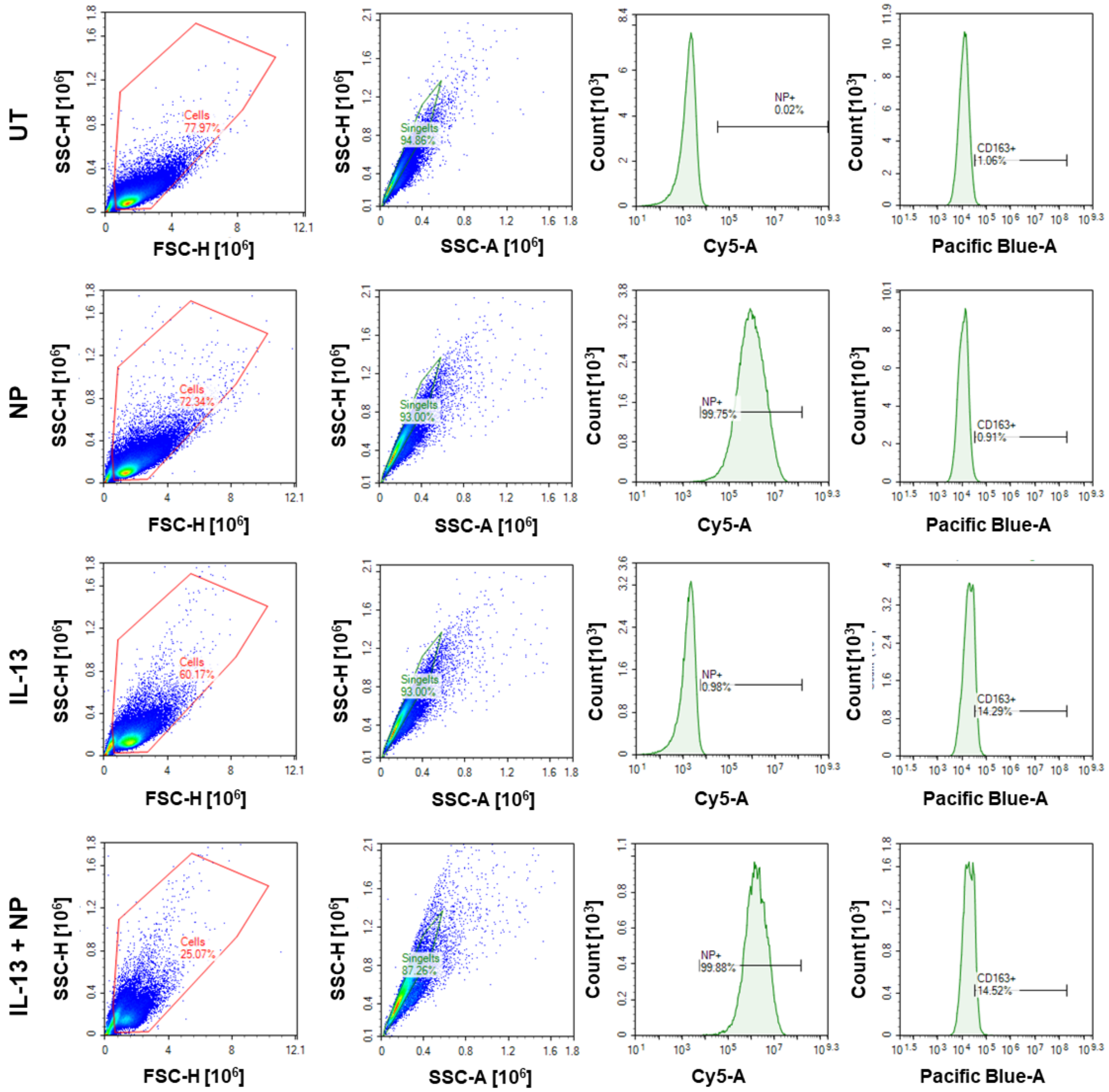

Supplemental Figure S11. Example flow cytometry plots of BMM samples following dosing and 72 hrs incubation with PEGDA(-)50, IL-13, or PEGDA(-)50 and IL-13. Anti-Inflammatory marker CD163 highlighted here.

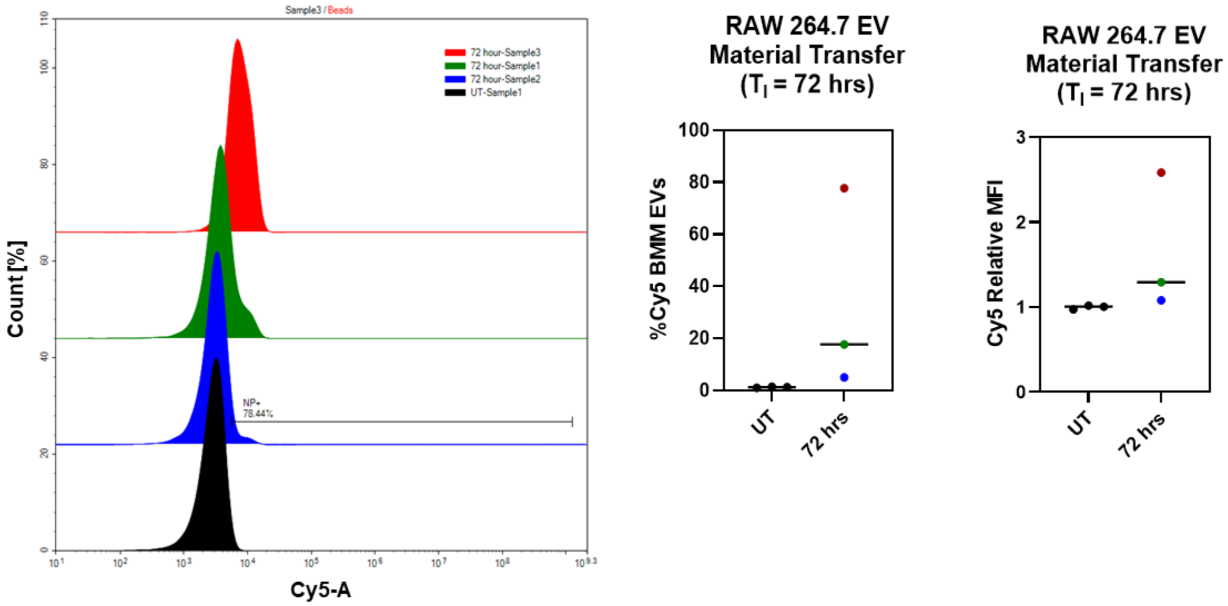

Supplemental Figure S12. Anti-CD63 capture bead flow cytometry analysis, sample histograms, relative Cy5 MFI, and %Cy5+ BMM EVs, in PEGDA(-)50 amplified EVs as a marker for NP material transfer for T_I = 72 hrs.

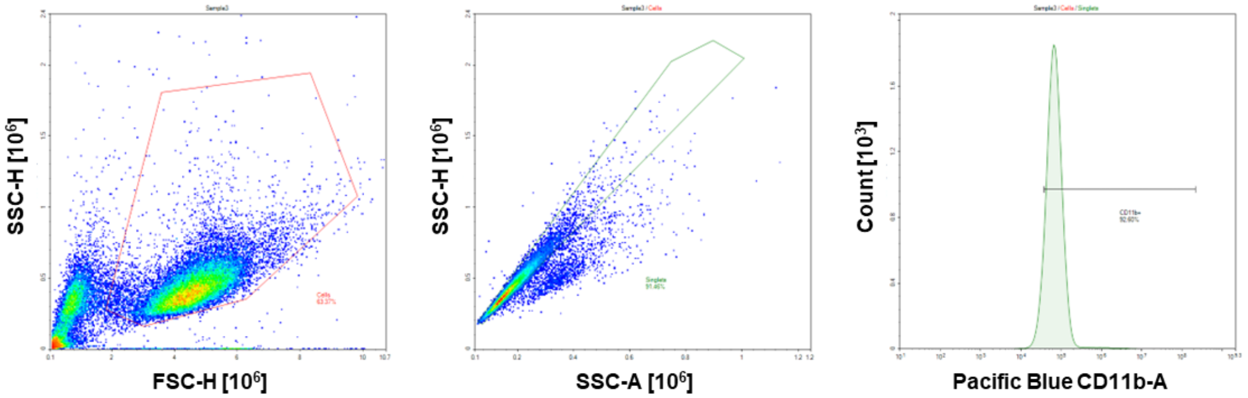

Supplemental Figure S13. Example flow gating for BMMs, with BMM marker CD11b+ highlighted.
